# Mucosal Immunity and Protective Efficacy of Intranasal Inactivated Influenza Vaccine Is Improved by Chitosan Nanoparticle Delivery in Pigs

**DOI:** 10.3389/fimmu.2018.00934

**Published:** 2018-05-02

**Authors:** Santosh Dhakal, Sankar Renu, Shristi Ghimire, Yashavanth Shaan Lakshmanappa, Bradley T. Hogshead, Ninoshkaly Feliciano-Ruiz, Fangjia Lu, Harm HogenEsch, Steven Krakowka, Chang Won Lee, Gourapura J. Renukaradhya

**Affiliations:** ^1^Food Animal Health Research Program, Department of Veterinary Preventive Medicine, The Ohio State University, Wooster, OH, United States; ^2^Department of Comparative Pathobiology, College of Veterinary Medicine, Purdue University, West Lafayette, IN, United States; ^3^Department of Veterinary Biosciences, The Ohio State University, Columbus, OH, United States

**Keywords:** swine influenza virus, chitosan nanoparticles, mucosal immune response, intranasal vaccination, pigs

## Abstract

Annually, swine influenza A virus (SwIAV) causes severe economic loss to swine industry. Currently used inactivated SwIAV vaccines administered by intramuscular injection provide homologous protection, but limited heterologous protection against constantly evolving field viruses, attributable to the induction of inadequate levels of mucosal IgA and cellular immune responses in the respiratory tract. A novel vaccine delivery platform using mucoadhesive chitosan nanoparticles (CNPs) administered through intranasal (IN) route has the potential to elicit strong mucosal and systemic immune responses in pigs. In this study, we evaluated the immune responses and cross-protective efficacy of IN chitosan encapsulated inactivated SwIAV vaccine in pigs. Killed SwIAV H1N2 (δ-lineage) antigens (KAg) were encapsulated in chitosan polymer-based nanoparticles (CNPs-KAg). The candidate vaccine was administered twice IN as mist to nursery pigs. Vaccinates and controls were then challenged with a zoonotic and virulent heterologous SwIAV H1N1 (γ-lineage). Pigs vaccinated with CNPs-KAg exhibited an enhanced IgG serum antibody and mucosal secretory IgA antibody responses in nasal swabs, bronchoalveolar lavage (BAL) fluids, and lung lysates that were reactive against homologous (H1N2), heterologous (H1N1), and heterosubtypic (H3N2) influenza A virus strains. Prior to challenge, an increased frequency of cytotoxic T lymphocytes, antigen-specific lymphocyte proliferation, and recall IFN-γ secretion by restimulated peripheral blood mononuclear cells in CNPs-KAg compared to control KAg vaccinates were observed. In CNPs-KAg vaccinated pigs challenged with heterologous virus reduced severity of macroscopic and microscopic influenza-associated pulmonary lesions were observed. Importantly, the infectious SwIAV titers in nasal swabs [days post-challenge (DPC) 4] and BAL fluid (DPC 6) were significantly (*p* < 0.05) reduced in CNPs-KAg vaccinates but not in KAg vaccinates when compared to the unvaccinated challenge controls. As well, an increased frequency of T helper memory cells and increased levels of recall IFNγ secretion by tracheobronchial lymph nodes cells were observed. In summary, chitosan SwIAV nanovaccine delivered by IN route elicited strong cross-reactive mucosal IgA and cellular immune responses in the respiratory tract that resulted in a reduced nasal viral shedding and lung virus titers in pigs. Thus, chitosan-based influenza nanovaccine may be an ideal candidate vaccine for use in pigs, and pig is a useful animal model for preclinical testing of particulate IN human influenza vaccines.

## Introduction

Influenza is caused by influenza A virus (IAV) of *Orthomyxoviridae* family. It is an economically important disease in the global pig industry (1, 2). Virulent swine IAV (SwIAV) infection leads to acute febrile respiratory disease which is often complicated with secondary bacterial infections (3). SwIAV increases its genetic diversity through frequent antigenic drift and antigenic shift. So far, H1N1, H1N2, and H3N2 subtypes are the major SwIAV circulating in pig populations (4). Since epithelial cells lining the porcine respiratory tract bear receptors for both avian and human IAVs, pigs can be infected with IAV from different hosts, and this event favors genetic assortment and adaptation of novel influenza strains of zoonotic and even pandemic potential (5). The pandemic H1N1 virus of 2009 and the more recent “H3N2 variant” virus in the USA are recent examples of swine-origin IAVs which cause infection and resultant pulmonary disease in humans (6, 7). Controlling influenza in pigs through vaccination serves dual benefits by protecting economic loss in swine industry and preventing possible public health risk that these reassorted SwIAVs pose for humans.

Swine influenza vaccines are commercially available. These are multivalent whole-inactivated virus (WIV) vaccines that are administered intramuscularly (IM) (8). The WIV vaccines provide protection against homologous virus infections but do not induce adequate heterologous immunity against constantly evolving IAVs that develop by point mutation(s) (8, 9). Moreover, the IM route used for WIV vaccines does not elicit adequate mucosal immune responses which are essential for providing cross-protective immunity against multitude of variant IAVs (10, 11). Intranasal (IN) vaccine that targets mucosal immune system of the respiratory tract can be a useful alternative to the current IM influenza vaccines used in pigs. Nasal mucosal vaccination not only induces strong protective immune responses at mucosal sites in the respiratory tract but also enhances immunity at distal mucosal and systemic sites (12, 13).

Biodegradable and biocompatible polymer-based nanoparticle (NP) formulation(s) provide an innovative strategy of vaccine antigen delivery to mucosal sites (14). Particulate vaccines facilitate antigen uptake by professional antigen-presenting cells (APCs), maintain slow and sustained antigen release, prevent the antigen(s) from undesirable enzymatic degradation, and potentiate the levels of protective immunity (14, 15). Different types of NPs are investigated for IN delivery of influenza vaccine antigens. For example, IN immunization in mice using liposome-based DNA and subunit influenza nanovaccines are shown to elicit mucosal, cellular, and humoral immune responses (16, 17). Poly(lactic-co-glycolic) acid (PLGA) NP-entrapped highly conserved H1N1 influenza virus peptides administered IN enhances the epitope-specific T cell response and protective efficacy in pigs (18). Ferritin-based IN influenza nanovaccine is shown to enhance mucosal secretary IgA and T cell response and confers homo- and heterosubtypic protection in mice (19). In our previous study, killed SwIAV antigen (KAg) encapsulated in PLGA polymer-based NP and delivered IN induced a robust cross-reactive cell-mediated immune response associated with a significant clearance of challenge heterologous virus from the lungs of pigs (20). In another study, the encapsulation of KAg in polyanhydride polymer-based NP also enhanced the cross-reactive cell-mediated immune response against SwIAV (21). However, both PLGA and polyanhydride polymer-based NP SwIAV vaccines used IN in these studies failed to elicit mucosal IgA and systemic IgG antibody responses, most likely due to their biased ability to induce strong T helper 1 (Th1) but not T helper 2 (Th2) responses. This Th1-biased response failed to reduce the nasal virus shedding in pigs (20, 21).

In the present study, we used chitosan, a natural mucoadhesive polymer derived NPs (CNPs) for the encapsulation of SwIAV KAg (CNPs-KAg) and performed a heterologous vaccine challenge trial in nursery pigs. Due to its cationic nature, chitosan binds readily to mucosal surfaces. Chitosan also possesses adjuvant properties, a feature which promotes immune activation (22). Previous studies have shown that CNPs form an attractive platform for mucosal vaccine delivery. For example, live Newcastle disease virus (NDV) encapsulated in CNPs and delivered through oral and IN route in chickens induced a higher secretary IgA antibody response in intestinal mucosa and enhanced the protective efficacy against highly virulent NDV strain challenge infection (23). Similarly, influenza subunit/split virus vaccine delivered in CNPs by IN route improves systemic and mucosal antibody and cell-mediated immune responses in mice (24–26). Hence, we hypothesized that IN delivery of chitosan-based nanovaccine would enhance both mucosal antibody and cellular immune responses and provide better protective immunity against SwIAV in pigs compared to soluble IN-inactivated vaccine. Our results demonstrated that CNPs-KAg IN vaccination improved mucosal IgA response in the entire respiratory tract and also elicited cell-mediated immune response against different subtypes of SwIAV, resulting in a reduced nasal viral shedding and an infectious virus burden in the pulmonary parenchyma.

## Materials and Methods

### SwIAV Propagation and Inactivation

Field isolates of IAVs A/Swine/OH/FAH10-1/10 (H1N2) (27), A/Swine/OH/24366/2007 (H1N1) (28), and A/Turkey/OH/313053/2004 (H3N2) (29) were propagated in Madin–Darby canine kidney (MDCK) cells. The H1N2 A/Swine/OH/FAH10-1/10 (H1N2-OH10) was used for CNPs-KAg vaccine preparation and H1N1 A/Swine/OH/24366/2007 (H1N1-OH7) was used for the virulent virus challenge infection. The H3N2 A/Turkey/OH/313053/2004 (H3N2-OH4) was used together with H1N2-OH10 and H1N1-OH7 for *ex vivo* cross-reactive immune analysis. The H1N2-OH10 vaccine virus and H1N1-OH7 challenge virus are heterologous to each other with 77% HA gene identity, whereas H3N2-OH4 virus, originally isolated from turkeys, is heterosubtypic to other two SwIAVs with HA gene identity of 63% (27–29). For vaccine preparation, cell culture fluid of H1N2-OH10 virus grown in MDCK cells was harvested and subjected to sucrose gradient ultracentrifugation. The virus pellet was suspended in phosphate-buffered saline (PBS), titrated for infectious virus titer, and inactivated by using binary ethyleneimine (Sigma, MO, USA) as described previously (20).

### Preparation of Chitosan-Based Nanovaccine and *In Vitro* Characterization

Chitosan NPs-loaded-killed SwIAV antigen (KAg) (CNPs-KAg) formulation was prepared by the ionic gelation method as described previously (23, 30–32) with some modifications. Briefly, 1.0% (w/v) low-molecular weight chitosan polymeric (Sigma, MO, USA) solution was prepared in an aqueous solution of 4.0% acetic acid under magnetic stirring until the solution became clear. The chitosan solution was sonicated; pH was adjusted to 4.3 and filtered *via* a 0.44-μm syringe filter. Five milliliters of 1.0% chitosan solution was added to 5.0-mL deionized water and incubated with 3.0-mg SwIAV KAg dissolved in 1.0 mL 3-(N-morpholino) propanesulfonic acid (MOPS) buffer at pH 7.4. Consequently, 2.5 mL of 1.0% (w/v) tripolyphosphate (TPP) (Sigma, MO, USA) dissolved in 2.5-mL deionized water was added into the chitosan polymer solution with continuous magnetic stirring at room temperature (RT) (22^°^C). The formulated SwIAV nanovaccine was centrifuged at 10,000 rpm for 10 min, dispersed in MOPS buffer at pH 7.4, lyophilized with a cryoprotectant, and stored at −80^°^C.

Particle size and zeta potential of empty and vaccine antigen-loaded NPs were measured after dispersion in PBS (pH 7.4) and stored at 4^°^C for at least 30 h by dynamic light-scattering (DLS) method using a zeta-sizer coupled with an MPT-2 titrator (Malvern) as described previously (33). During each vaccination, CNPs-KAg were freshly prepared and used. The morphology of NPs was obtained by using the cold field emission Hitachi S-4700 scanning electron microscope (SEM) (20). Briefly, the powder form of NPs was loaded on to aluminum stubs and coated with platinum prior to examination under the microscope. Protein loading efficiency in CNPs-KAg was estimated indirectly by determining the difference between the initial amount of protein used for loading CNPs and the protein left in the supernatant (23). *In vitro* protein release profile in CNPs-KAg suspended in PBS for up to 15 days was estimated and expressed as the cumulative percentage release of SwIAV antigen at each time point as described previously (20). In brief, CNPs-KAg suspended in 500 μL PBS (pH 7.4) in triplicate in eppendorf tubes was incubated at 37^°^C in a revolving roller apparatus. At indicated time point, tubes were centrifuged, supernatant collected, and pellet was resuspended in fresh 500 μL PBS. Protein released on to the supernatant was estimated by micro-BCA protein assay kit (Thermo Scientific, MA, USA) and expressed as the percentage of cumulative protein released over the initial amount at time zero in particles.

### *In Vitro* Uptake of CNPs-KAg by APCs

Peripheral blood mononuclear cells (PBMCs) isolated from 9- to 10-week-old pigs were used for the *in vitro* antigen uptake study. Cells were suspended in enriched-Roswell Park Memorial Institute medium and seeded on to 1 million cells/well in 96-well cell culture plates. After overnight incubation at 37^°^C in 5% CO_2_, unattached cells were removed. The attached monocyte/macrophage cells were treated with SwIAV KAg or CNPs-KAg containing the antigen at 10 μg/mL concentration for 10, 30, and 150 min. After the indicated period of incubation, the cells were fixed with 80% acetone, stained with IAV nucleoprotein-specific antibody (CalBioreagents, CA, USA) followed by Alexa Fluor 488 conjugated goat anti-mouse IgG antibody (Life technologies, OR, USA). Cells were evaluated under fluorescent microscope (Olympus IX70) and photomicrographs were taken (20×). For evaluation of SwIAV antigen uptake from CNPs-KAg-treated porcine monocyte/macrophages prepared from three pigs, PBMCs separately were incubated in 48-well plates seeded with 2 × 10^6^ cells per well overnight as described above. Cells were treated with KAg or CNPs-KAg at SwIAV antigen concentration 10 μg/mL for 10, 30, and 150 min. A positive control was MDCK cells infected with SwIAV H1N2-OH10 at multiplicity of infection (MOI) 1 for 12 h. After the indicated period of treatment, cells were fixed using 1% paraformaldehyde (PFA), permeabilized, and stained with IAV nucleoprotein-specific antibody (CalBioreagents, CA, USA) followed by treatment with goat anti-mouse IgG Alexa Flour 488 conjugated secondary antibody. We acquired 50,000 events in BD Aria II flow cytometer (BD Biosciences, CA, USA) and the data analyzed by using the FlowJo software (Tree Star, OR, USA).

### *In Vitro* Generation and Stimulation of Porcine Dendritic Cells (DCs)

Porcine monocyte-derived dendritic cells (MoDCs) were prepared from PBMCs isolated from seven pigs as described previously (34) with few modifications. Briefly, 25 million PBMCs per mL were seeded in each well of six-well culture plates. After overnight incubation at 37^°^C in a 5% CO_2_ incubator, non-adherent cells were discarded and adhered cells were treated with GM-CSF (25 ng/mL) and interleukin (IL-4) (10 ng/mL) cytokines. Half of the culture media was replaced on every third day. On day 7, the plate was centrifuged at 2,000 RPM at 4^°^C for 5 min and the supernant was harvested gently, and the generated immature MoDCs were stimulated in the same plate without seeding into fresh plates with medium only, LPS control (10 μg/mL), KAg (10 μg/mL), and CNPs-KAg containing 10 μg/mL of KAg for 48 h. The culture supernatant was harvested, and the levels of innate, pro-inflammatory and Th1 cytokines, IFN-α, TNF-α, IL-1β, IL-12, IL-6, and IL-10 were estimated by ELISA as described previously (35).

### Experimental Design

Cesarean-delivered colostrum-deprived and bovine colostrum-fed influenza antibody-free Large White-Duroc crossbred piglets were raised in our BSL2 animal facility at OARDC. Piglets at 4 weeks of age (male and female) were randomly assigned into one of the three experimental groups and kept in separate isolation rooms (Table 1). The first IN vaccination was performed at 5 weeks of age and the second IN booster vaccination at 8 weeks of age. All piglets receiving virulent SwIAV were challenged at 10 weeks of age. Separate groups of pigs were vaccinated IN with DMEM (Gibco) or with 1 × 10^7^ TCID_50_ equivalent of KAg or CNPs-KAg suspended in 2 mL DMEM by IN mist as described previously (20). The challenge infection was done using heterologous H1N1-OH7 SwIAV (6 × 10^6^ TCID_50_) in 2 mL, divided into 1 mL administered IN and 1 mL intratracheally as described previously (18, 20, 21).

**Table 1:** Experimental design showing different vaccine groups.

Experimental groups	Pig no.	First vaccination (DPV 0)	Second vaccination (DPV 21)	Day of challenge (DPV 35/DPC 0)
Unvaccinated	3	DMEM	DMEM	H1N1-OH7
KAg	4	Inactivated H1N2-OH10	Inactivated H1N2-OH10	H1N1-OH7
CNPs-KAg	5	Inactivated H1N2-OH10 encapsulated in chitosan nanoparticle (CNP)	Inactivated H1N2-OH10 encapsulated in CNP	H1N1-OH7

Serum samples were collected at days post-vaccination (DPV) 21 and 35. The rectal temperatures were recorded daily from day post-challenge (DPC) 0 onward, and nasal swab samples were collected at DPC 0, DPC 4, and DPC 6. Pigs were euthanized at DPC 6, and serum and bronchoalveolar lavage (BAL) fluid were collected. During necropsy, lungs were examined for macroscopic pneumonic lesions and scored as described previously (20). Lung lysates were prepared by homogenization of 1.0 g of lung tissue collected from the right apical lobe (20). Nasal swabs, sera, BAL fluid, and lung lysate samples were stored at −80^°^C until processed for antibody and virus titration. The PBMCs were isolated from blood at DPV 35/DPC 0 and DPC 6 (20). Mononuclear cells were harvested from tracheobronchial lymph nodes (TBLN-MNCs) at DPC 6 as described previously (35).

### Antibody Titration

Hemagglutination inhibition (HI) antibody titers against IAVs H1N1-OH7, H1N2-OH10, and H3N2-OH4 in sera and BAL fluid samples were determined as described previously (20). The SwIAV-specific IgG and IgA antibodies in nasal swabs, sera, BAL fluids, and lung lysates were determined by ELISA (20). Briefly, 96-well plates (Greiner bio-one, NC, USA) were coated overnight with respective pre-titrated IAV antigen (5 μg/mL) and blocked with 5% skim milk powder containing 0.05% Tween-20 for 2 h at RT. After washing, fivefold dilutions of nasal swab, serum, BAL fluid, and lung lysate samples in PBS containing 2.5% skim milk powder and 0.05% Tween-20 were added to marked duplicate wells, incubated for 2 h at RT, washed, and horse radish peroxidase-conjugated goat anti-pig IgA (Bethyl Laboratories Inc., TX, USA) or goat anti-pig IgG (KPL, MD, USA) was added. Finally, the antigen and antibody interaction were detected by using 1:1 mixture of peroxidase substrate solution B and TMB peroxidase substrate (KPL, MD, USA). The reaction was stopped using 1.0 M phosphoric acid, and optical density was measured at 450 nm using Spectramax microplate reader (Molecular devices, CA, USA).

### Antigen-Specific Cell Proliferation Assay

The PBMCs isolated at DPV 35/DPC 0 were cultured together with H1N1-OH7, H1N2-OH10, or H3N2-OH4 SwIAV at 0.1 MOI and incubated at 37^°^C in 5% CO_2_ incubator for 72 h. Antigen-specific lymphocyte proliferation was determined by using the cell titer 96 aqueous non-radioactive proliferation assay kit (Promega, WI, USA). The cell proliferative response was compared among groups using lymphocyte stimulation index values as described previously (20).

### Cytokine ELISA

The PBMCs isolated at DPC 0 and TBLN-MNCs at DPC 6 were cultured with H1N2-OH10, H1N1-OH7, or H3N2-OH4 SwIAV at 0.1 MOI. After 72 h of stimulation, the supernatant was collected and interferon gamma (IFNγ) secretion was determined by ELISA as described previously (20). Similarly, the production of interleukin-6 (IL-6) in BAL fluid collected at DPC 6 was determined by ELISA (35).

### Virus Titration

Viral titers contained in nasal swabs and BAL fluids were determined in a 10-fold dilution of the samples in DMEM containing TPCK-trypsin (1 μg/mL). The samples were transferred to quadruplicate 96-well cell culture plate wells containing overnight cultured monolayers of MDCK cells and incubated for 72 h, 37^°^C, 5.0% CO_2_. Cells were fixed with acetone and immunostained with IAV nucleoprotein-specific antibody (#M058, CalBioreagents, CA, USA) followed by Alexa Fluor 488 conjugated goat anti-mouse IgG (H + L) antibody (Life technologies, OR, USA). Virus replication in cells was determined by using immunofluorescence technique as described previously (20).

### Histopathology of Lungs

For histopathological analysis of pulmonary tissues, 10% formalin-inflated apical, cardiac, and diaphragmatic lobes were collected and further emulsion-fixed in 10% neutral buffered formalin. Five-micrometer sections of formalin-fixed, paraffin-embedded apical, cardiac, and diaphragmatic lung lobes were stained with hematoxylin and eosin (H&E) as previously described (20). The H&E-stained tissue sections were examined for microscopic changes of interstitial pneumonia, peribronchial and perivascular accumulation of mononuclear cells, bronchial exudates, and epithelial changes related to influenza infection. All these parameters were scored by a board-certified veterinary pathologist (SK) who was not provided with any vaccination history of pig groups in a scale of 0 (no change compared from normal) to 3 (marked changes from normal) as described previously (20).

### Flow Cytometry

The PBMCs isolated at DPC 0 and TBLN-MNCs at DPC 6 were immunostained for T lymphocyte subset phenotyping as described previously (20). Antibodies used in the flow cytometry were anti-porcine CD3 (Southernbiotech, AL, USA), CD4α (Southernbiotech, AL, USA), CD8α (Southernbiotech, AL, USA), and CD8β (BD Biosciences, CA, USA). Briefly, the cells were blocked with 2% pig serum in fluorescence-activated cell sorting (FACS) buffer and surface labeled with pig lymphocyte-specific purified, biotin or fluorochrome-conjugated antibodies or their respective antibody isotypes. Cells were fixed using 1% PFA, washed, suspended in FACS buffer, and acquired using BD Aria II flow cytometer (BD Biosciences, CA, USA). Data analysis was done using FlowJo software (Tree Star, OR, USA).

### Quantitative Reverse Transcription PCR (RT-qPCR)

Total RNA was extracted from PBMCs at DPC 0 and TBLN at DPC 6 using TRIzol reagent (Invitrogen, CA, USA) as per the manufacturer’s instructions. NanoDrop™ 2000c Spectrophotometer (Thermo Fisher Scientific, MA, USA) was used to determine the concentration and purity of RNA. cDNA was prepared from 1 μg of total RNA using the QuantiTect Reverse Transcription Kit (AIAGEN). Primers of housekeeping gene (β actin) and target genes (T-bet and GATA-3) used in this experiment were described previously (36). The mRNA expression was analyzed by 7500 Real-Time qPCR system (Applied Biosystems, CA, USA) using the qScript™ One-Step SYBR Green qRT-PCR kit, Low ROX™ (Quantabio, MA, USA). The target gene expression level was normalized with housekeeping gene levels, and the fold change was determined by comparative 2^−ΔΔ^*^C^*T method (37).

### Statistical Analysis

Statistical analysis was performed by using non-parametric Kruskal–Wallis test followed by Dunn’s *post hoc* test using the software GraphPad Prism 5 (GraphPad Software, Inc., CA, USA). Pig rectal temperature data were analyzed by repeated measure ANOVA using Friedman test followed by Dunn’s pairwise comparison. Cytokine data (Figure 2) between two groups were analyzed by Mann–Whitney test. A *p*-value of less than 0.05 was considered to be statistically significant. The infectious virus titer was determined using Reed and Muench method. Data were presented as the mean ± SEM of three to five pigs except for the HI titers which were expressed as the geometric mean with 95% confidence interval.

## Results

### Characterization of CNPs-KAg Vaccine Candidate

**Figure 1:**
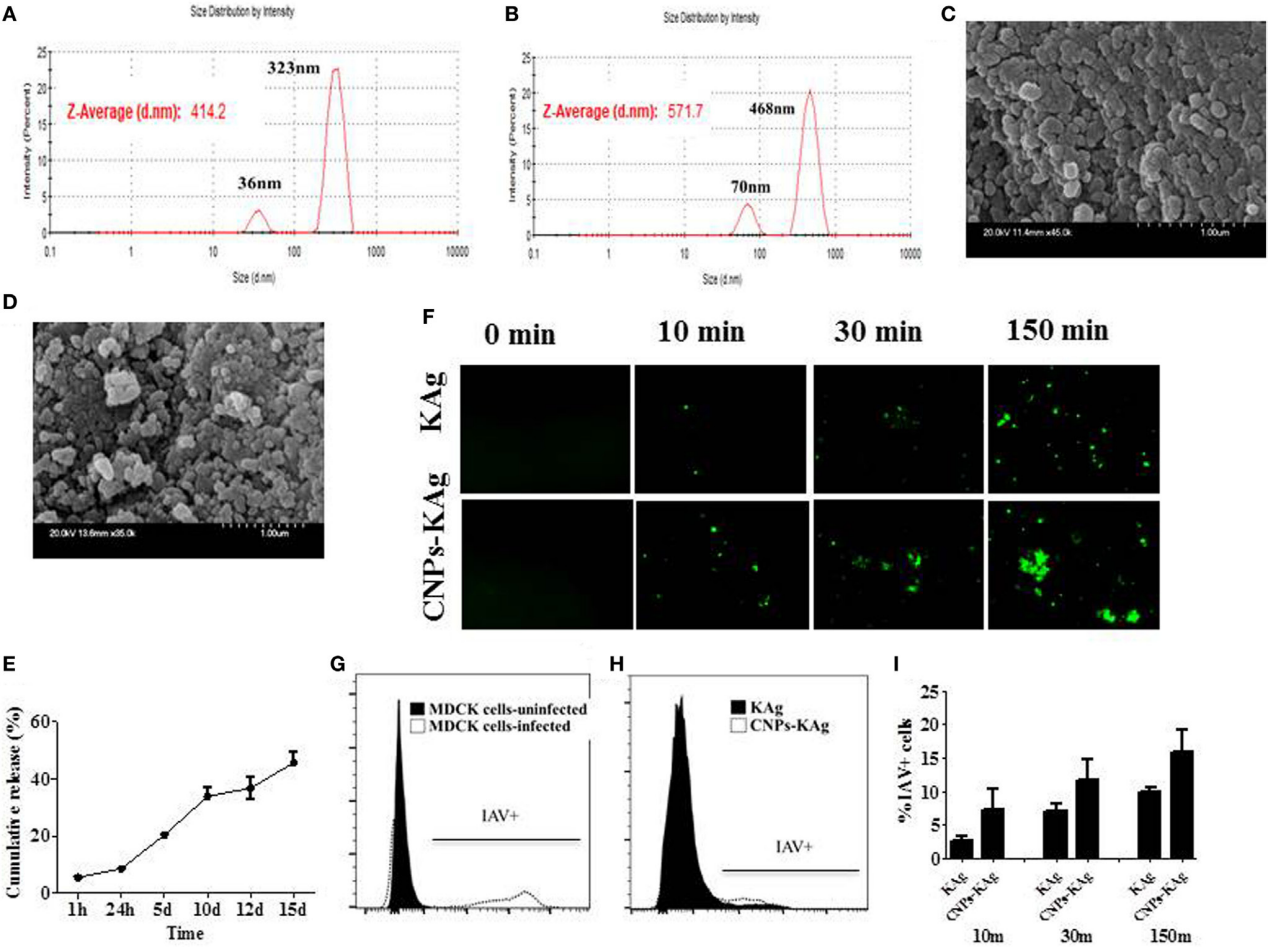
*In vitro* characteristics of chitosan nanoparticles (CNPs)-KAg. Diameter of **(A)** empty CNPs and **(B)** swine influenza A virus (SwIAV)-killed antigen (KAg)-loaded CNPs (CNPs-KAg) determined by dynamic light scattering. Scanning electron microscope (SEM) images of **(C)** empty CNPs and **(D)** CNPs-KAg. **(E)** Release of KAg from CNPs-KAg suspended in phosphate-buffered saline over a period of 15 days. **(F)** Uptake of soluble SwIAV KAg or CNPs-KAg formulation by monocytes/macrophages at indicated time points determined by fluorescent microscopy (Olympus, IX70, 20× magnifications). The frequency of monocytes/macrophages uptaken SwIAV KAg treated with soluble antigen or CNPs-KAg determined by flow cytometry: **(G)** SwIAV-infected Madin-Darby canine kidney (MDCK) cells as positive control; **(H)** a representative picture of SwIAV KAg or CNPs-KAg uptake by porcine monocytes/macrophages after 150 min treatment; and **(I)** percentage of cells with internalized SwIAV antigen at 10, 30, and 150 min treatment.

The encapsulation efficiency of SwIAV KAg in chitosan nanovaccine formulation was 67%. This result was comparable to the encapsulation efficiency of chitosan NPs entrapped with *Salmonella* outer membrane protein antigens (70%) (Renu et al., 2018, manuscript under review). As determined by DLS, the average size of the empty (Figure 1A) and antigen-loaded (Figure 1B) NPs was 414.2 and 571.7 nm, respectively. Empty NPs showed two peaks at 36 (~10%) and 323 nm (~90%) with polydispersity index (PDI) of 0.39. Likewise, antigen-loaded NPs also had two peaks at 70 (~15%) and 468 nm (~85%) with PDI of 0.60. Data show that the CNPs-KAg were polydispersed in nature. SEM analysis showed that the morphology of the empty NPs was spherical with a smooth surface (Figure 1C), while antigen-loaded NPs had a relatively rough and irregular surface (Figure 1D). The surface charge of empty and antigen-loaded chitosan NPs was +1.88 and +1.69 mV, respectively. We observed 6% burst release, i.e., surface-associated antigen release during the first 1 h, and on an average, 9% of antigen was released after 24 h of incubation. Further, a slow and sustained release of antigen was observed with a cumulative release of approximately 46% after 15 days (Figure 1E).

To determine whether chitosan encapsulation of KAg enhances the uptake of antigen by APCs, we prepared monocyte/macrophages from PBMCs and allowed for interaction with KAg or CNPs-KAg and stopped the reaction at three different time points. Internalization of CNPs-KAg vaccine by monocytes/macrophages was observed within 10 min of treatment indicated by a higher number of influenza-specific fluorescent signals compared to KAg treatment (Figure 1F). Further, the uptake of CNPs-KAg was substantially increased after 30 and 150 min post treatment compared to control KAg-treated cells. We also performed flow cytometry analysis of monocyte/macrophages treated with KAg or CNPs-KAg to determine the frequency of specific uptake of influenza antigens in APCs. MDCK cells infected with SwIAV H1N2-OH10 were used as positive control (Figure 1G) and a representative picture of flow cytometry analysis of KAg or CNPs-KAg uptake by monocyte/macrophages is also shown (Figure 1H). In soluble KAg-treated cells, an average of 2.7, 7.1, and 10.1% cells positive for influenza antigen, and in CNPs-KAg-treated cells, 7.2, 11.7, and 16% cells with uptaken influenza antigen after 10, 30, and 150 min of incubation were noticed, respectively (Figure 1I). These data clearly demonstrated that CNPs-KAg was efficiently internalized by pig APCs better than soluble antigens.

### CNPs-KAg Formulation Induced the Secretion of Cytokines by Porcine MoDCs *In Vitro*

**Figure 2:**
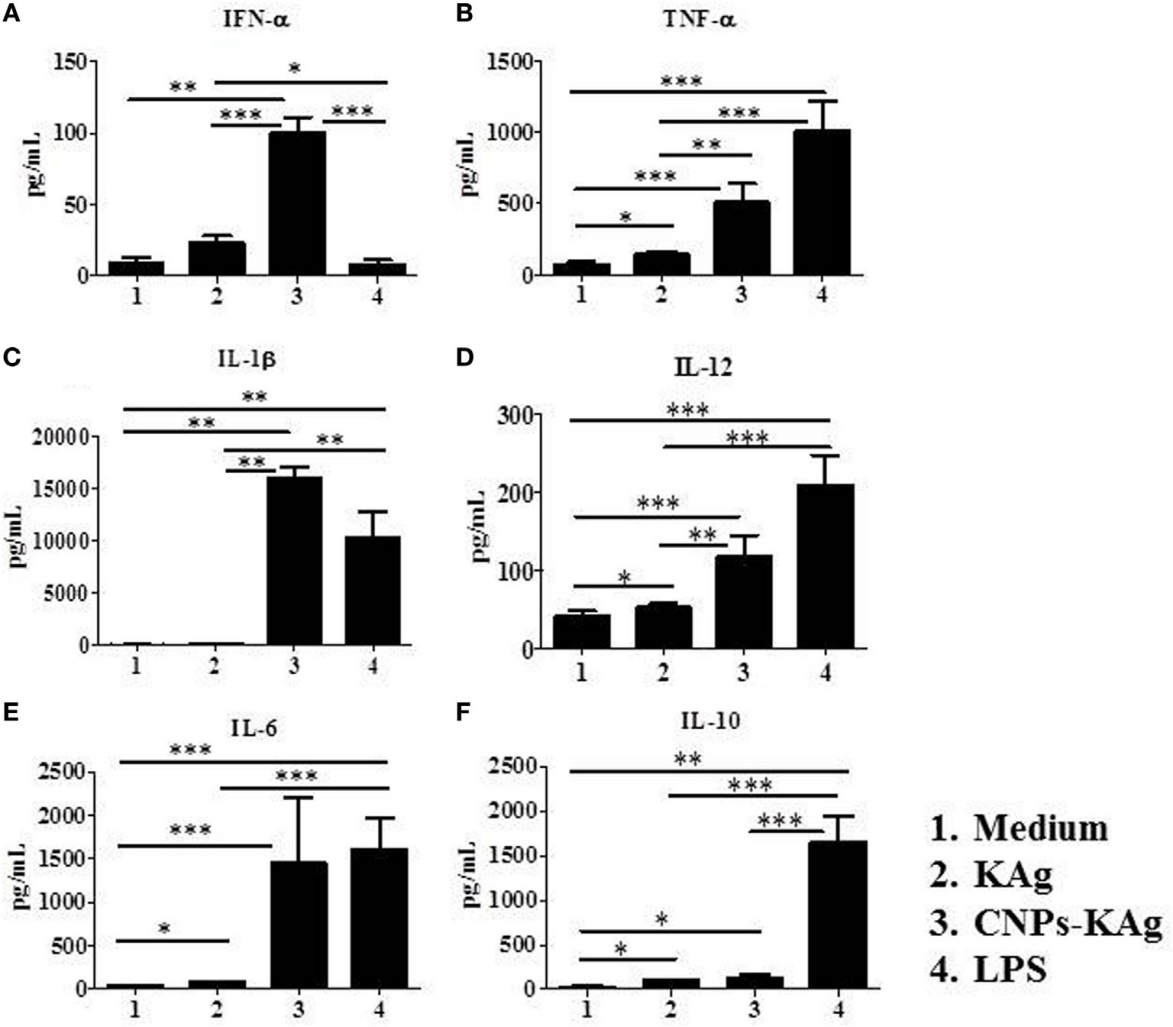
Production of innate, pro-inflammatory and T helper 1 cytokines by porcine MoDCs treated for 48 h with medium, KAg, chitosan nanoparticles (CNPs)-KAg or LPS control. Levels of cytokines **(A)** IFN-α, **(B)** TNF-α, **(C)** interleukin (IL)-1β, **(D)** IL-12, **(E)** IL-6, and **(F)** IL-10 were estimated in stimulated cell culture supernatant by ELISA. Data represent mean value of seven pig-derived DCs ± SEM. Statistical analysis between two groups was carried out using Mann–Whitney test. Asterisk refers to statistical significant difference between the indicated two pig groups. *p < 0.05, **p < 0.01, and ***p < 0.001.

In order to elucidate the adjuvant property of chitosan NPs in porcine APCs, we treated porcine MoDCs with medium and LPS as control to compare the effect of soluble KAg and CNPs-KAg treatment in inducing the secretion of different cytokines. As expected, the medium control cells had very little secretion of all the detected cytokines, while LPS treatment induced the production of all the analyzed cytokines except IFN-α (Figures 2A–F). Cells treated with KAg secreted significantly higher levels of pro-inflammatory cytokines TNF-α (Figure 2B) and IL-6 (Figure 2E) and Th1 cytokine IL-12 (Figure 2D) compared to medium control. In DCs treated with CNPs-KAg, the production of innate IFN-α (Figure 2A), TNF-α (Figure 2B), IL-1β (Figure 2C), and IL-12 (Figure 2D) was significantly higher in CNPs-KAg-treated compared to that in soluble KAg-treated cells. In CNPs-KAg-treated cells, the production of IL-6 (Figure 2E) and Th2 cytokine IL-10 (Figure 2F) was higher than medium control cells, but not significantly higher compared to KAg-treated cells. Our *in vitro* DCs treatment data suggest that chitosan nanovaccine formulation has a potent adjuvant effect on porcine DCs.

### CNPs-KAg Vaccine Augmented the IAV-Specific Mucosal Antibody Response in the Respiratory Tract of Pigs

**Figure 3:**
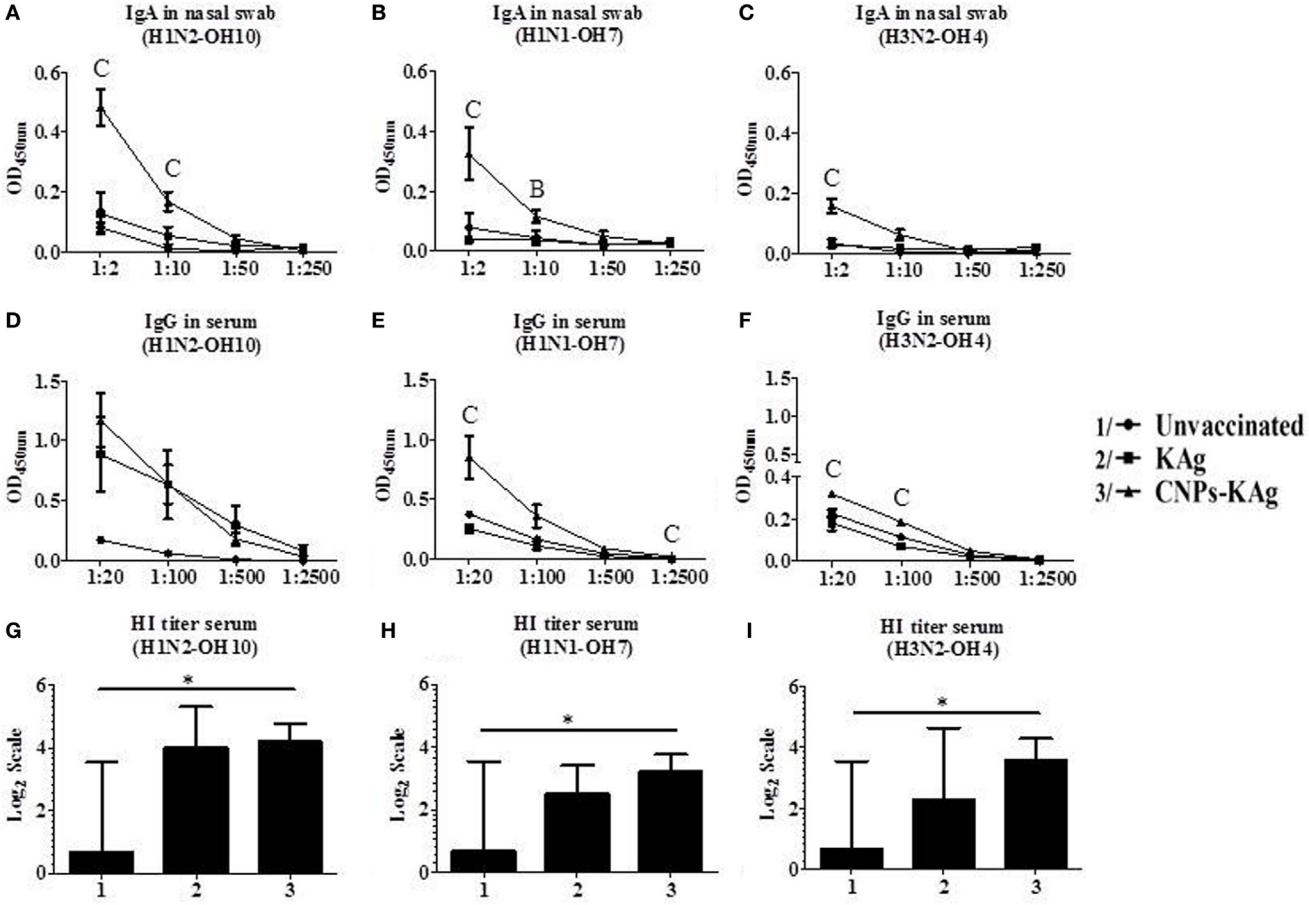
Antibody response after chitosan nanoparticles (CNPs)-KAg prime-boost vaccination at day post-vaccination 35/day post-challenge 0 (DPV 35/DPC 0) in pigs. Mucosal secretory IgA antibody response in nasal swab, systemic IgG antibody, and hemagglutination inhibition (HI) titers in serum samples against **(A,D,G)** H1N2-OH10, **(B,E,H)** H1N1-OH7, and **(C,F,I)** H3N2-OH4 influenza A virus (IAVs). Data represent the mean value of three to five pigs ± SEM. Statistical analysis was carried out using Kruskal–Wallis test followed by Dunn’s *post hoc* test. Asterisk refers to statistical significant difference between the indicated two pig groups (*p < 0.05). In antibody dilution curves **(A–F)**, A, B, and C refer to significant difference between unvaccinated vs KAg vaccinates, unvaccinated vs CNPs-KAg vaccinates, and KAg vs CNPs-KAg vaccinates, respectively, at the indicated dilution.

**Figure 4:**
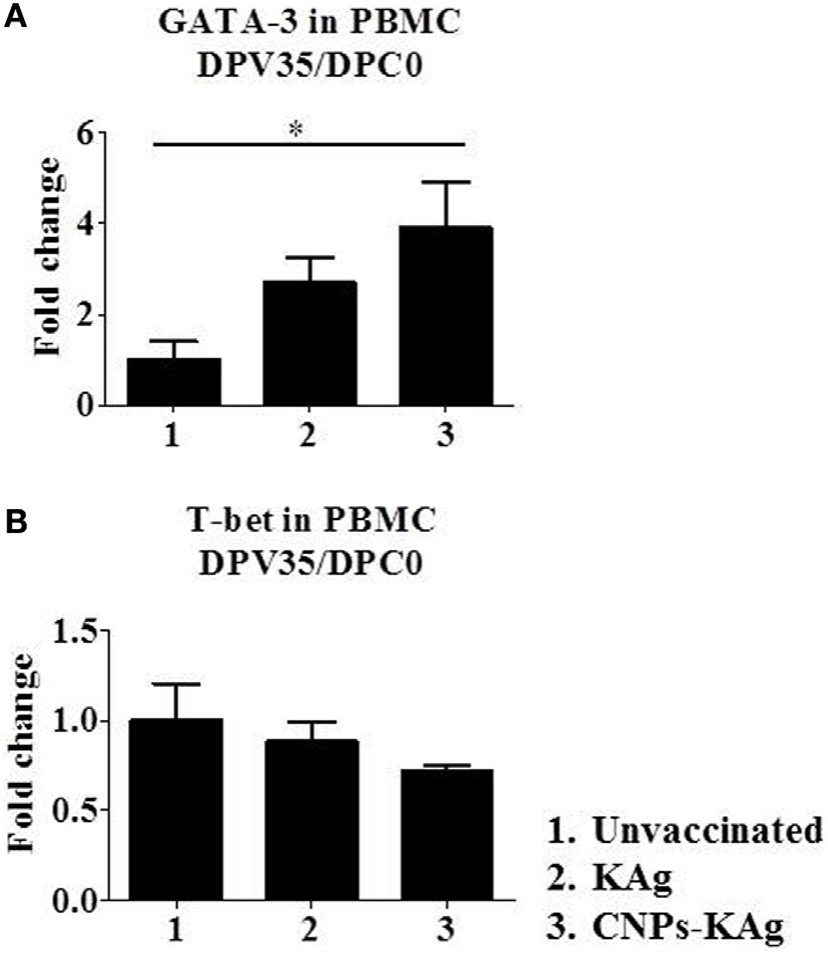
Expression of T helper 1 (Th1) and T helper 2 (Th2) response inducing specific transcription factors after prime-boost vaccination in pigs. The expression of **(A)** Th2 transcription factor GATA-3 and **(B)** Th1 transcription factor T-bet in peripheral blood mononuclear cells of pigs at day post-vaccination (DPV) 35/day post-challenge (DPC) 0 were determined by quantitative reverse transcription PCR (qRT-PCR). Data represent the mean value of three to five pigs ± SEM. Statistical analysis was carried out using Kruskal–Wallis test followed by Dunn’s *post hoc* test. Asterisk refers to the statistical significant difference between the indicated two pig groups. *p < 0.05.

Secretary IgA antibody levels in nasal swab samples collected after IN prime-boost vaccination at DPV 35/DPC 0 were significantly higher (*p* < 0.05) in CNPs-KAg-vaccinated pigs compared to those in pigs receiving soluble KAg when tested against the homologous H1N2-OH10 (Figure 3A), heterologous H1N1-OH7 (Figure 3B), and heterosubtypic H3N2-OH4 IAVs (Figure 3C). A significant difference in antibody response was observed between CNPs-KAg and KAg vaccinates in serial twofold diluted nasal swab samples (Figures 3A–C). These data suggest that the CNPs-KAg IN delivery induced an enhanced cross-reactive mucosal secretary IgA antibody response in pigs. Specific IgG antibody response in sera after prime-boost vaccination in KAg-vaccinated pigs against the vaccine virus was comparable to the CNPs-KAg vaccine group (Figure 3D). A significantly higher (*p* < 0.05) cross-reactive IgG response was observed in CNPs-KAg vaccinates against heterologous H1N1-OH7 (Figure 3E) and heterosubtypic H3N2-OH10 (Figure 3F) IAVs compared to KAg-vaccinated animals. In CNPs-KAg-vaccinated pig sera, IAV-specific HI antibody titers against H1N2-OH10 (Figure 3G), H1N1-OH7 (Figure 3H), and H3N2-OH4 (Figure 3I) were significantly higher (*p* < 0.05) compared to mock pig group. The HI titers in CNPs-KAg vaccinates were around twofold higher compared to KAg vaccinates against heterologous (Figure 3H) and heterosubtypic (Figure 3I) IAVs, but the data were not statistically significant (*p* > 0.05). The expression of Th2-specific transcription factor GATA-3 mRNA in PBMCs of pigs at DPV 35/DPC 0 was 4- and 1.5-fold higher in CNPs-KAg-vaccinated pigs compared to that in unvaccinated control (*p* < 0.05) and KAg-vaccinated pigs (*p* > 0.05) (Figure 4A). The expression of Th1-specific transcription factor T-bet in PBMCs was not significantly different among the pig groups (Figure 4B).

**Figure 5:**
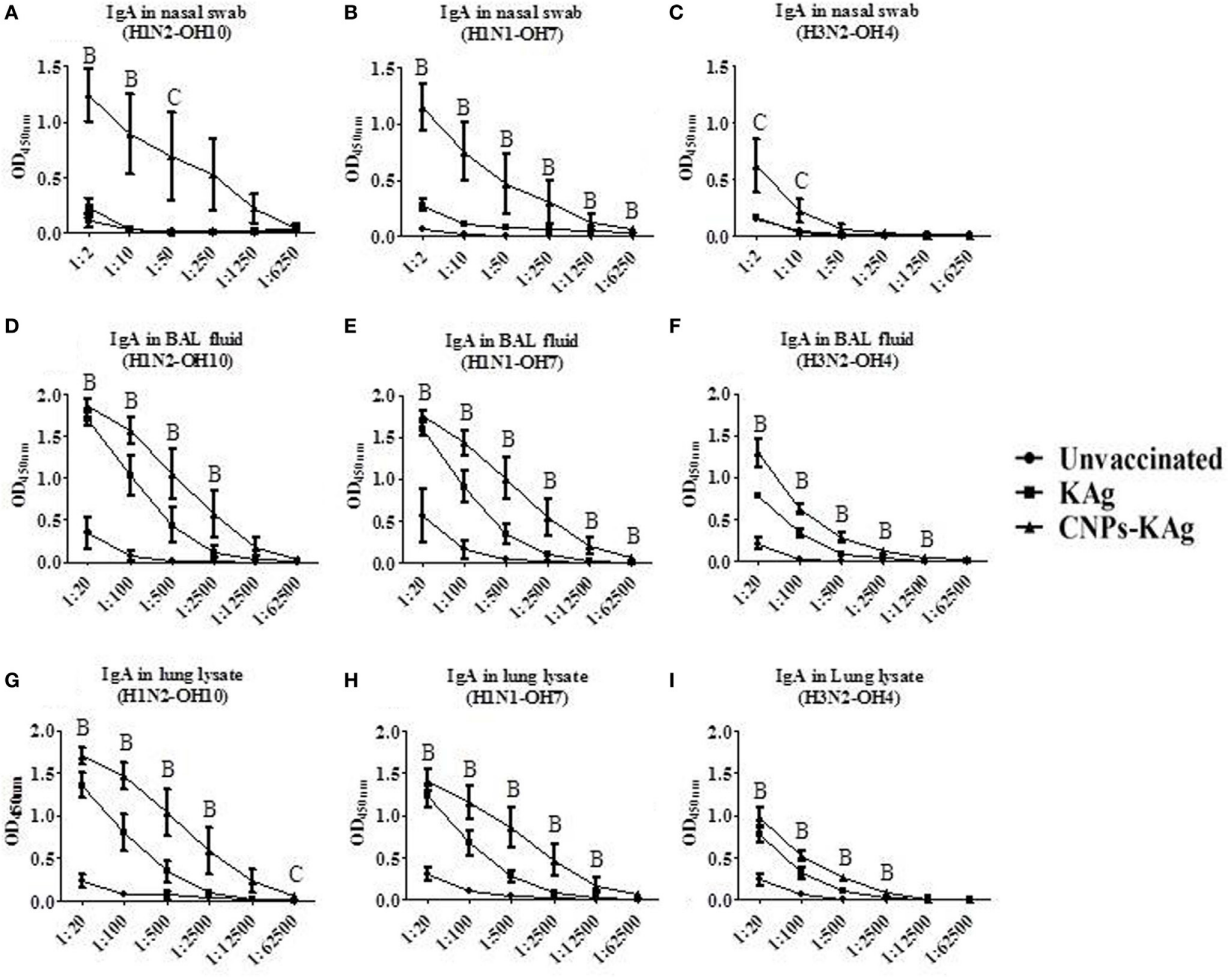
Mucosal IgA antibody response in the respiratory tract of pigs vaccinated with chitosan nanoparticles (CNPs)-KAg at day post-challenge 6. Specific IgA antibody response in nasal swab, bronchoalveolar lavage (BAL) fluid, and lung lysate samples against H1N2-OH10 **(A,D,G)**, H1N1-OH7 **(B,E,H)**, and H3N2-OH4 **(C,F,I)** influenza A virus (IAVs). Data represent the mean value of three to five pigs ± SEM at all indicated dilutions. Statistical analysis was carried out using Kruskal–Wallis test followed by Dunn’s *post hoc* test where A, B, and C refer to significant difference (*p* < 0.05) between unvaccinated vs KAg vaccinates, unvaccinated vs CNPs-KAg vaccinates, and KAg vs CNPs-KAg vaccinates, respectively, at the indicated dilution.

**Figure 6:**
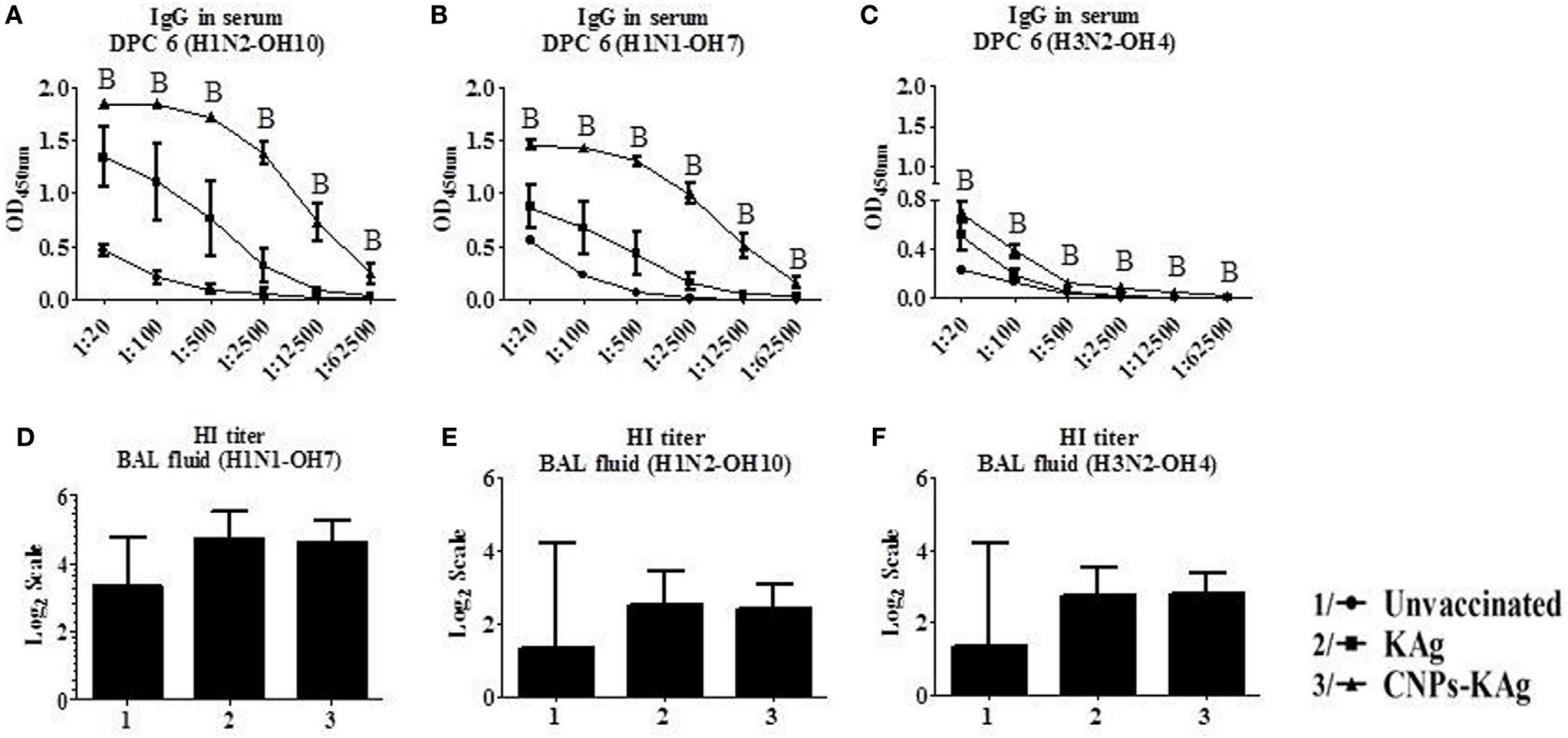
Serum IgG response and bronchoalveolar lavage (BAL) fluid hemagglutination inhibition (HI) antibody titers in pigs vaccinated with chitosan nanoparticles (CNPs)-KAg at day post-challenge 6. Specific IgG antibody response in serum and BAL fluid HI titers against H1N2-OH10 **(A,E)**, H1N1-OH7 **(B,D)**, and H3N2-OH10 **(C,F)** influenza A virus (IAVs). Data represent the mean value of three to five pigs ± SEM. Statistical analysis was carried out using Kruskal–Wallis test followed by Dunn’s *post hoc* test. Asterisk refers to the statistical significant difference between the indicated two pig groups (*p < 0.05). In antibody dilution curves **(A–C)**, A, B, and C refers to significant difference between unvaccinated vs KAg vaccinates, unvaccinated vs CNPs-KAg vaccinates, and KAg vs CNPs-KAg vaccinates, respectively, at the indicated dilution.

The mucosal IgA response in pigs post-challenge at DPC 6 was determined, and the data indicate that specific IgA in CNPs-KAg-vaccinated pig group was significantly higher (*p* < 0.05) compared to unvaccinated-challenged animals and remarkably higher compared to KAg-vaccinated and -challenged animals against all three IAV subtypes in nasal swabs (Figures 5A–C), BAL fluids (Figures 5D–F), and lung lysates (Figures 5G–I). These data indicated the secretion of robust mucosal IgA antibody in the upper respiratory tract (nasal swabs), lower respiratory tract (BAL fluids), and lung parenchyma (lung lysates) of pigs. Similarly, systemic IgG antibody response in serum at DPC 6 was also enhanced in the CNPs-KAg vaccinates compared to that in unvaccinated (*p* < 0.05) and KAg-vaccinated and virus-challenged animals (Figures 6A–C). However, HI antibody titers in BAL fluid at DPC 6 were comparable between KAg and CNPs-KAg vaccinates (Figures 6D–F).

### CNPs-KAg Vaccine Enhanced Systemic-Specific Cell-Mediated Immune Response Against IAVs

**Figure 7:**
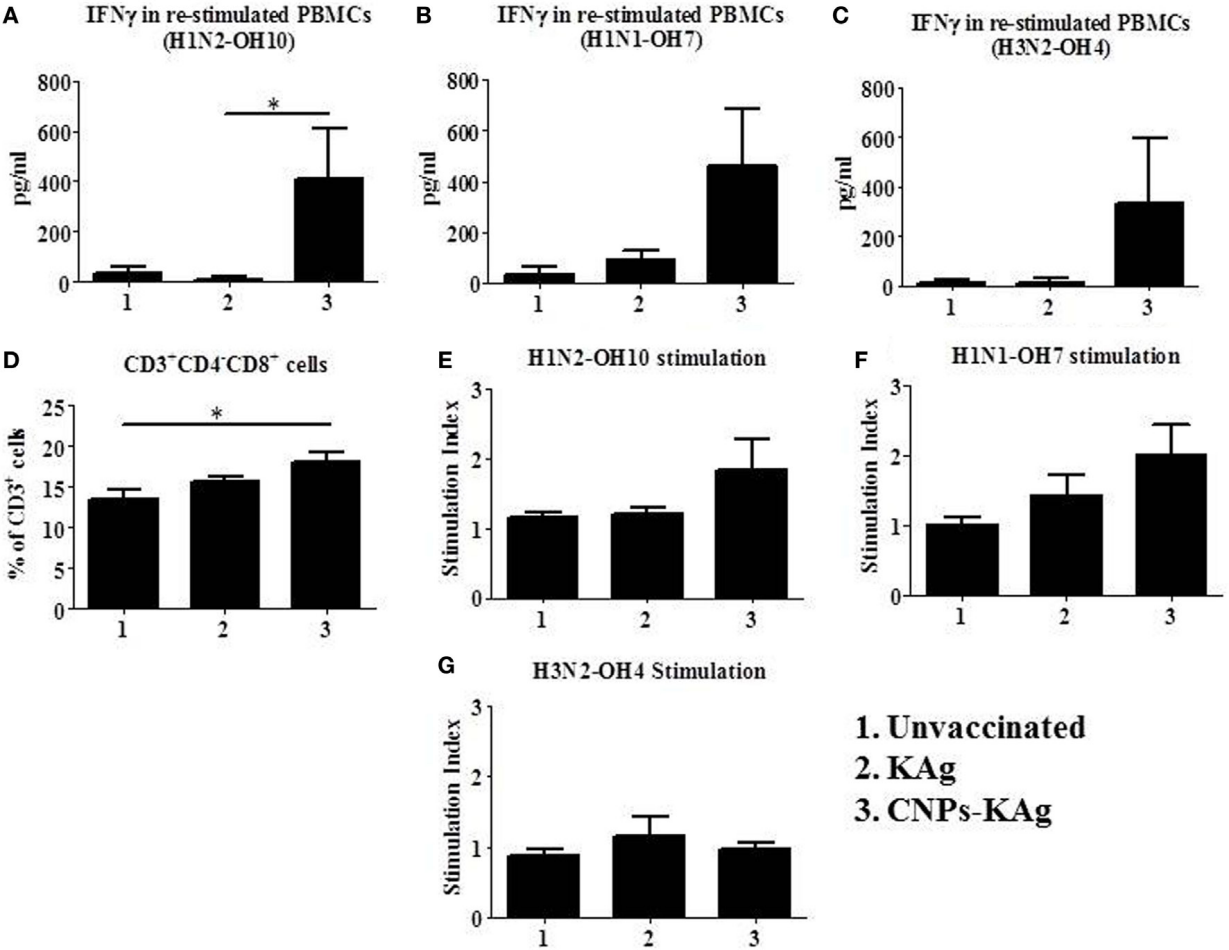
Cell-mediated immune response after prime-boost vaccination was enhanced in chitosan nanoparticles (CNPs)-KAg-vaccinated pigs at pre-challenge days post-vaccination (DPV) 35/day post-challenge (DPC) 0. Peripheral blood mononuclear cells (PBMCs) isolated from blood were stimulated with different variant influenza A virus (IAVs). IFNγ secretion in the culture supernatant and antigen-specific lymphocyte proliferation was determined after 72 h of stimulation with **(A,E)** H1N2-OH10, **(B,F)** H1N1-OH7, and **(C,G)** H3N2-OH4 IAVs. **(D)** Flow cytometry analysis of PBMCs showed enhanced frequency of CTLs (CD3^+^CD4^−^CD8αβ^+^) in CNPs-KAg-vaccinated pigs. Data represent the mean value of three to five pigs ± SEM. Statistical analysis was carried out using Kruskal–Wallis test followed by Dunn’s *post hoc* test. Asterisk refers to the statistical significant difference between the indicated two pig groups (*p < 0.05).

To understand the role of chitosan delivered IAV nanovaccine in the induction of specific cell-mediated immune response after IN vaccinations, isolated PBMCs at DPV 35 were restimulated with H1N2-OH10, H1N1-OH7, and H3N2-OH4 viruses. The harvested cell culture supernatants were analyzed for IFNγ secretion, and significantly higher (*p* < 0.05) levels of IFNγ in homologous H1N2-OH10 virus restimulated CNPs-KAg compared to soluble KAg-vaccinated pigs were observed (Figure 7A). Though not statistically significant, the IFNγ recall response in heterologous and heterosubtypic viruses restimulated cells was noticeably of higher levels in CNPs-KAg vaccinates than in KAg-vaccinated pigs (Figures 7B,C). The average IFNγ amounts in CNPs-KAg vaccine group against H1N1-OH7 and H3N2-OH4 viruses were 463 and 332 pg/mL compared to 91 and 16 pg/mL in KAg vaccinates, respectively (Figures 7B,C). We also performed phenotyping of PBMCs isolated at DPC 0 by flow cytometry. The frequency of cytotoxic T cells (CTLs) in CNPs-KAg-vaccinated pigs (average: 18.1%) was higher compared to that in KAg-vaccinated (average: 15.7%, *p* > 0.05) and unvaccinated pigs (average: 13.4%, *p* < 0.05) (Figure 7D). This finding is consistent with enhanced IFNγ response in CNPs-KAg-vaccinated pigs, as activated CTLs are one of the major T cell subsets which secrete high levels of antiviral cytokine IFNγ. In addition, in PBMCs at DPC 0, virus-specific cell proliferation was detected in an increased trend upon restimulation with homologous (Figure 7E) and heterologous (Figure 7F), but not with heterosubtypic (Figure 7G) viruses in CNPs-KAg-vaccinated pigs. Overall, these data suggested the presence of superior cross-reactive effector memory lymphocyte response in pigs induced by chitosan encapsulation of inactivated SwIAV antigen.

### CNPs-KAg Vaccine Reduced the Inflammatory Changes in the Lungs of Virulent and Heterologous Virus-Challenged Pigs

**Figure 8:**
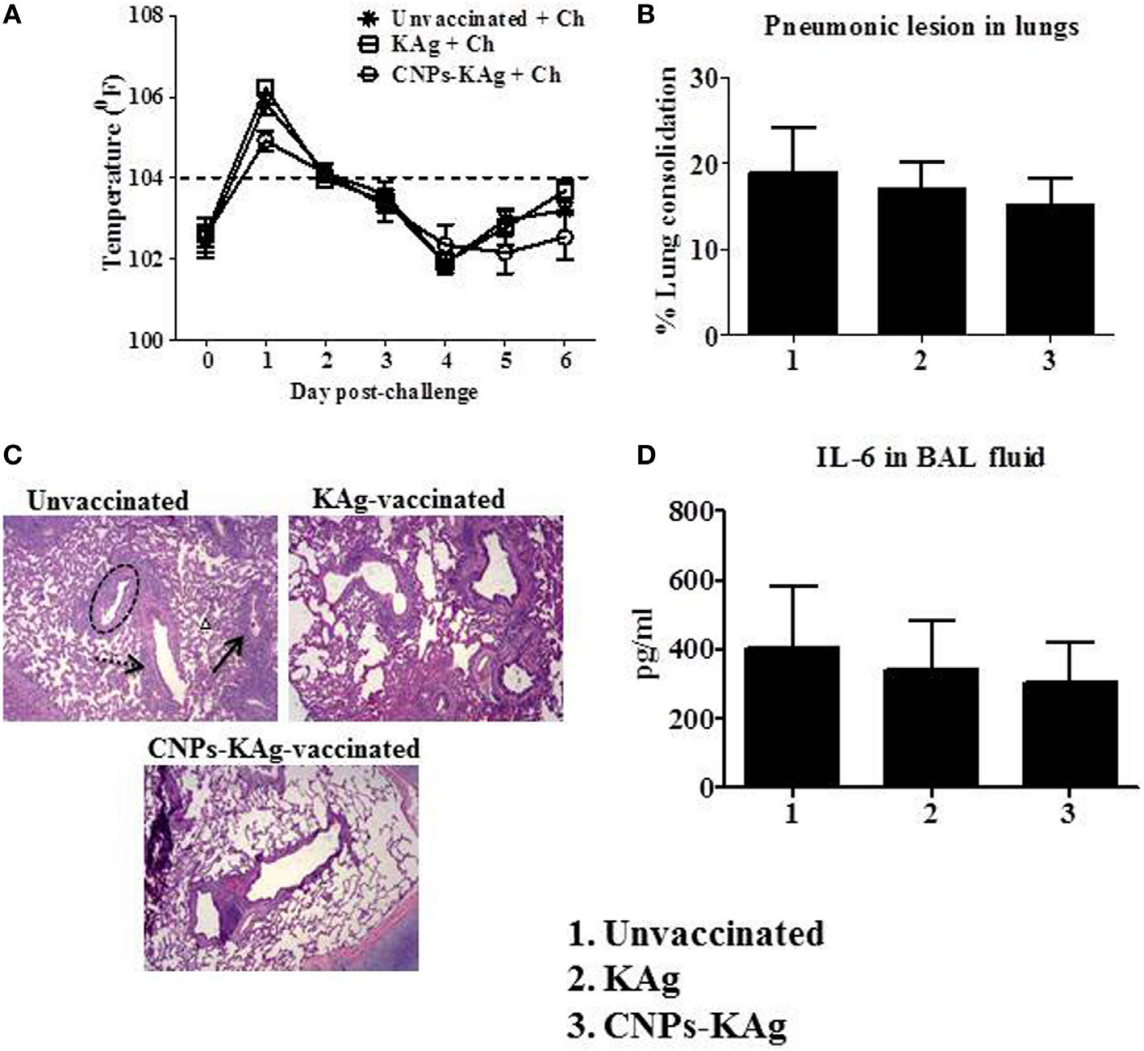
Clinical and pathological changes in pigs vaccinated with chitosan nanoparticles (CNPs)-KAg post-challenge. **(A)** Rectal temperature was recorded daily after challenge until the day of necropsy. **(B)** Gross pneumonic lesions in lungs determined at day post-challenge (DPC) 6. **(C)** Representative Hematoxylin and eosin (H&E)-stained lung pictures showing bronchial exudates (dotted black circle), perivascular inflammation (black arrow), peribronchial inflammation (dashed black arrow), and interstitial pneumonia (small black triangle). **(D)** Secretion of cytokine interleukin (IL-6) in bronchoalveolar lavage (BAL) fluid. Data represent the mean value of three to five pigs ± SEM.

Rectal temperature of pigs was recorded daily post-challenge until euthanized. Pigs in all groups had fever (≥104^°^F) for the first 2 days after challenge. However, there was no statistical difference in temperature profile among the pig groups (Figure 8A). Macroscopic pulmonary lesions were scored for percent consolidation induced by influenza infection and observed lower pulmonary consolidation in CNPs-KAg vaccinates (mean score 15) compared to KAg (mean score 17) and unvaccinated animals (mean score 19) (Figure 8B), but the data were not statistically significant (*p* > 0.05). Microscopic pulmonary lesions were subjectively scored on H&E-stained lung sections where a score of 0 = no change from normal, 1 = minimal change from normal, 2 = moderate change from normal, and 3 = severe change from normal (Figure 8C). The mean scores of interstitial pneumonia (2, 1.6, and 0.8), peribronchial inflammation (2, 1.8, and 1.8), perivascular inflammation (1.6, 0.3, and 0.5), bronchial exudates (0.7, 0.1, and 0.2), and epithelial changes (0.3, 0.3, and 0.1) were observed in virus-challenged unvaccinated, KAg, and CNPs-KAg vaccinates, respectively. All the microscopic evaluation of pulmonary tissues was conducted by a board-certified veterinary pathologist. A moderate reduction in inflammatory changes was observed in both the vaccinated pig groups when compared to the lesion scores in the unvaccinated and challenged group. In particular, the interstitial pneumonia and epithelial changes were much reduced in CNPs-KAg group compared to those in soluble KAg-vaccinated pigs.

We also evaluated the levels of pro-inflammatory cytokine IL-6 secretion in the BAL fluid and observed relatively lower levels in CNPs-KAg-vaccinated pigs, consistent with the lower macroscopic and microscopic lung lesions (Figure 8D).

### CNPs-KAg Vaccine Enhanced the Mucosal Cellular Immune Response in the Tracheobronchial Lymph Nodes of Virulent and Heterologous Virus-Challenged Pigs

**Figure 9:**
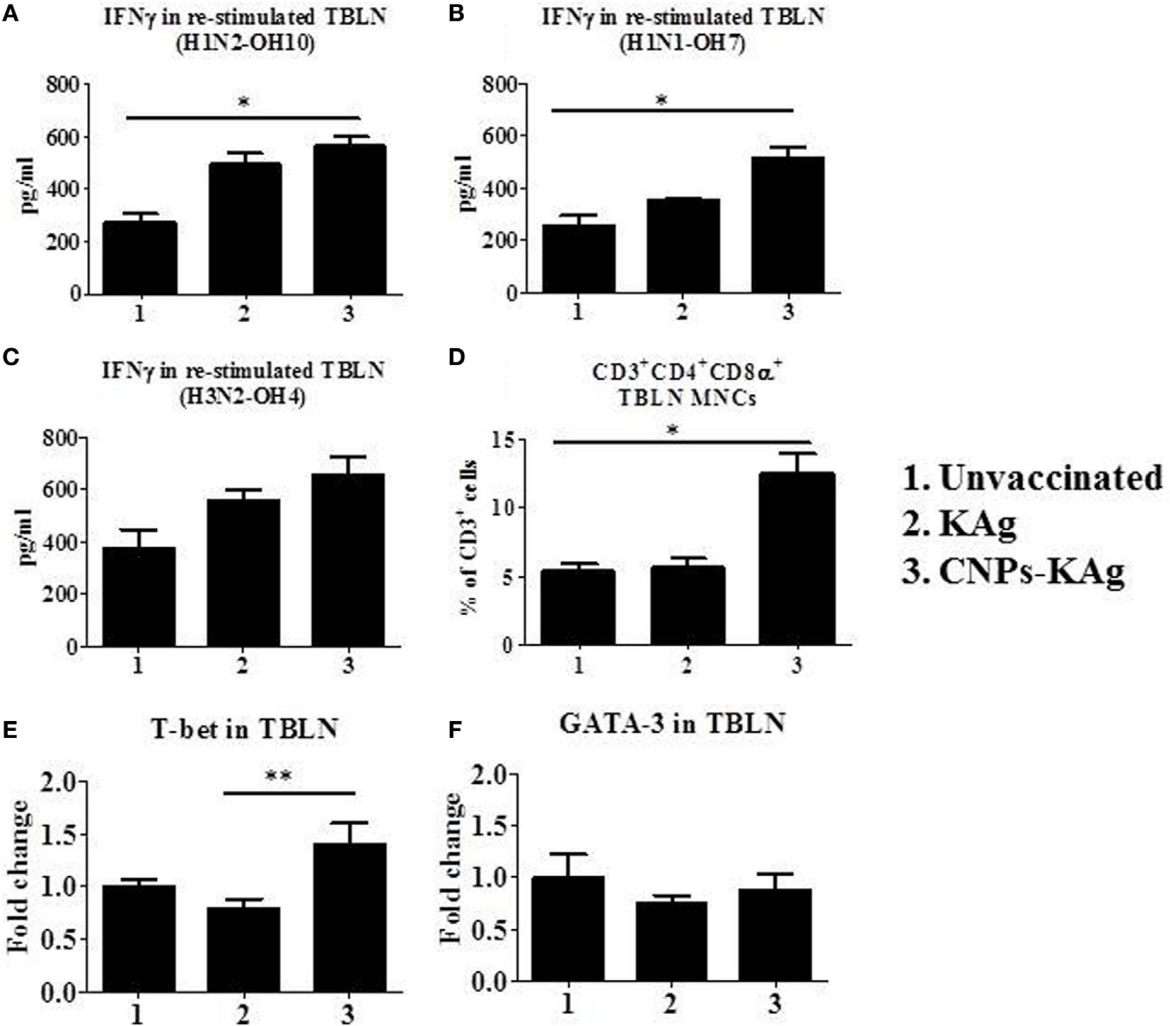
Cell-mediated immune response in TBLN-MNCs of pigs vaccinated with chitosan nanoparticles (CNPs)-KAg at day post-challenge (DPC) 6. TBLN-MNCs isolated on the day of necropsy were stimulated with different variant swine influenza A viruses (SwIAVs), and secreted IFNγ into the culture supernatant was measured by cytokine ELISA against **(A)** H1N2-OH10, **(B)** H1N1-OH7, and **(C)** H3N2-OH4 influenza A virus (IAVs). **(D)** The frequency of T helper (Th)/memory cells (CD3^+^CD4^−^CD8α^+^) in TBLN-MNCs of CNPs-KAg-vaccinated pigs was analyzed by flow cytometry. The expression of Th1 **(E)** and Th2 **(F)** transcription factors were also determined in TBLN at DPC 6. Data represent the mean value of three to five pigs ± SEM. Statistical analysis was carried out using Kruskal–Wallis test followed by Dunn’s *post hoc* test. Asterisk refers to the statistical significant difference between the indicated two pig groups (*p < 0.05).

In the CNPs-KAg-vaccinated pigs, the frequency of CTLs, IFNγ, and specific lymphocyte proliferation index values were augmented in PBMCs (Figure 7). The cell-mediated immune response in the lung draining TBLN was also examined in these vaccinated pigs. Our data demonstrated a significantly higher (*p* < 0.05) secretion of IFNγ by TBLN-MNCs restimulated with vaccine (H1N2-OH10) and challenge (H1N1-OH7) viruses in CNPs-KAg, but not in KAg-vaccinated compared to mock group (Figures 9A,B). Cells similarly stimulated with heterosubtypic (H3N2-OH4) IAV showed an increase in IFNγ secretion in CNPs-KAg-vaccinated pig group but this increase was not statistically significant (Figure 9C). We performed flow cytometry analysis of TBLN-MNCs isolated at DPC 6 and observed a significantly higher (*p* < 0.05) frequency of T helper/memory cells (CD3^+^CD4^+^CD8α^+^), one of the principle contributors of IFNγ production in pigs (18), in CNPs-KAg-vaccinated pig group compared to that in unvaccinated and challenged animals (Figure 9D). The expression of Th1 and Th2 transcription factors mRNA level in TBLN collected at DPC 6 was analyzed. Consistent with augmented cellular response in TBLN-MNCs of CNPs-KAg-vaccinated pigs, in frozen TBLN tissues mRNA, the expression of the Th1-specific transcription factor T-bet was significantly higher (*p* < 0.05) in CNPs-KAg compared to KAg vaccinates (Figure 9E). The expression of Th2 transcription factor GATA-3 mRNA was not increased in TBLN (Figure 9F).

### CNPs-KAg Vaccine Reduced Virus Shedding in the Nasal Cavity and Also Pulmonary Viral Titers in SwIAV-Challenged Pigs

**Figure 10:**
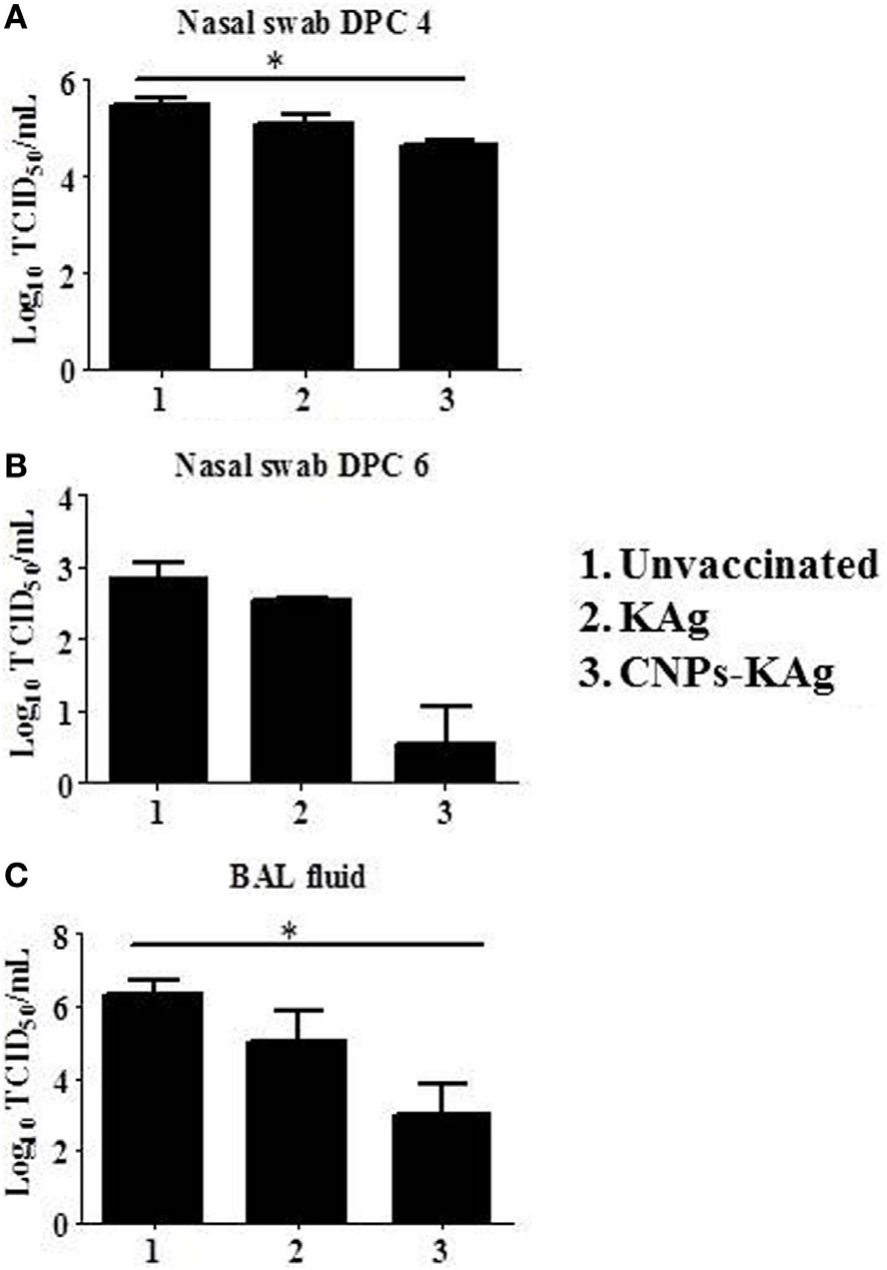
Infectious challenge swine influenza A virus (SwIAV) H1N1 titer in the respiratory tract of chitosan nanoparticles (CNPs)-KAg-vaccinated and influenza A virus (IAV)-challenged pigs. Titers of challenge SwIAV shedding through nostrils at **(A)** day post-challenge (DPC) 4 and **(B)** DPC 6, and in bronchoalveolar lavage (BAL) fluid at DPC 6 **(C)** determined by using cell culture technique. Data represent the mean value of three to five pigs ± SEM. Statistical analysis was carried out using Kruskal–Wallis test followed by Dunn’s *post hoc* test. Asterisk refers to the statistical significant difference between the indicated two pig groups (*p < 0.05).

We observed a significantly reduced (*p* < 0.05) challenge virus shedding at DPC 4 from the nasal passage of CNPs-KAg vaccinates compared to that of unvaccinated and challenged animals (Figure 10A). By DPC 6, infectious virus was detected in the nasal passage of only one of five pigs (20%) vaccinated with CNPs-KAg vaccine, while all pigs in KAg-vaccinated and unvaccinated groups were shedding virus ranging from 10^2.5^ to 10^3.3^ TCID_50_/mL (Figure 10B). The average virus titers in nasal swab at DPC 6 in unvaccinated, KAg-, and CNPs-KAg-vaccinated and -challenged pigs were 10^2.8^, 10^2.5^, and 10^0.5^ TCID_50_/mL, respectively (Figure 10B). Similarly, the virus titer in BAL fluid on DPC 6 was significantly reduced (*p* < 0.05) in CNPs-KAg but not in KAg group compared to unvaccinated virus challenge pigs (Figure 10C). The average virus titers in BAL fluid at DPC 6 in unvaccinated, KAg-, and CNPs-KAg-vaccinated and IAV-challenged pig groups were 10^6.3^, 10^5^, and 10^3^ TCID_50_/mL, respectively.

## Discussion

Chitosan is a natural polymer synthesized by deacetylation of chitin, one of the most abundant polysaccharides in nature (38). Chitosan forms an attractive excipient for drug and vaccine delivery as it bears biocompatible, biodegradable, mucoadhesive, polycationic, and immunomodulatory properties (38, 39). Chitosan is often coupled with TPP, a polyanion that helps in the encapsulation of the biochemical agents through ionotropic gelation. The chitosan and TPP (CS/TPP) NPs formulation in mice were shown to induce both cell-mediated (Th1) and humoral (Th2) immune responses when immunized through IN route against *Streptococcus equi* (40). Similarly, tetanus toxoid loaded in CS/TPP NPs IN delivered in rat was efficiently transported through the nasal epithelium, and in mice, it induced a long-lasting systemic and mucosal antibody response compared to soluble antigen (41). Mice immunized through IN route using CS/TPP-based influenza split virus vaccine were shown to induce a higher systemic and mucosal antibody response than soluble antigens and also enhanced the cell-mediated immune response indicated by an increased IFNγ-secreting cell frequency in spleen (25). Unlike the preparation of PLGA and polyanhydride NPs, the process of preparing CNPs does not need any organic solvents and thus involves a simple and mild procedure protecting sensitive biochemical agents including proteins and provides scope for the easy modification of particles (42–45).

In this study, we prepared chitosan-based influenza nanovaccine using TPP by ionotropic gelation technique. The resulting NPs were around 500 nm in diameter which is adequate for efficient uptake by APCs (18, 20, 21, 46). The size of NPs was slightly increased after antigen loading like reported earlier (47). But the surface charge of our NPs did not change much with or without antigen loading, and the charge (+2.84 mV) was comparable to NPs entrapped with NDV, which was also loaded in CS:TPP at 2:1 ratio formulation like our CNPs-KAg (23). We evaluated the stability of CNPs-KAg NPs suspended in physiological buffer until 30 h maintained at 4^°^C. For vaccination of pigs, CNPs-KAg was freshly prepared and maintained on ice until delivered IN (1–2 h) which ensured the stability of NPs vaccine.

For better stability and long-term storage of NP vaccines, the surface charge should be highly negative or positive (38, 48). But our CNPs-KAg NPs were polydispersed in nature and had a weak positive surface charge, suggesting that further improvements to our CNPs-KAg formulation are required through optimization of the ratio of CS:TPP:KAg to ensure better physicochemical properties. The optimal CS:TPP:KAg combination should yield a higher positive surface charge, monodispersed nature, relatively smaller size NPs (100–300 nm), and stable for a long time at different storage conditions. The encapsulation efficiency of KAg in chitosan NPs formulation was 67%, higher than the encapsulation efficiency of H1N2-OH10 KAg (~50–55%) in PLGA and polyanhydride NPs (20, 21). The higher encapsulation efficiency of vaccine antigens is desirable to reduce the cost of vaccine production. The protein release from CNPs-KAg was slower than previously reported similar CS/TPP NPs formulation, wherein close to 50% of NDV antigens were released from CNPs within the first 3 days (23). CNP encapsulation enhances the antigen uptake by APCs, increases the expression of activation markers, and secretion of pro-inflammatory cytokines by APCs (49). As expected, SwIAV antigens delivered in chitosan NPs were efficiently internalized by porcine APCs compared to soluble KAg, and importantly, induced the higher production of innate, pro-inflammatory, and Th1 cytokines compared to soluble KAg.

Induction of strong mucosal immunity is associated with an increased breadth of protective efficacy against influenza, and inactivated IM vaccines do not elicit high levels of antigen-specific mucosal IgA antibody response in the respiratory tract (10, 11). Moreover, IM influenza vaccines in pigs have a limitation of not being effective in the presence of maternal-derived antibody (MDA) (50). However, successful IN vaccination has a potential to overcome MDA interference because of the induction of robust local mucosal immunity in the respiratory tract with minimal MDA interference (51). Chitosan is an attractive polymer for IN immunization (22). It enhances the absorption of vaccine particles across the nasal epithelium (52). Further, when compared to aqueous chitosan solution, insulin-loaded CNPs (300–400 nm diameter) increased the nasal absorption of insulin (53). Due to the positive charge of chitosan, it can interact with anionic components such as sialic acid of glycoproteins on epithelial cell surfaces, thereby prolonging local retention time and decreasing antigen clearance on mucosal surfaces. In addition to its bioadhesive properties, chitosan enhances paracellular and intracellular transport of particulate antigens into the subepidermal space for optimal contact with APCs and other cells associated with immune responses (54, 55). In mice, the IN delivery of CNP-based hepatitis B vaccine enhances the mucosal IgA antibody response (56, 57). Other murine studies have shown that IN immunization with chitosan-based nanovaccine formulations induces robust mucosal and systemic antibody responses against *Pneumococcus* spp., *Diphtheria* spp., and *Bordetella* spp. (58–60).

An influenza subunit vaccine coadministered IN with chitosan delivery system enhanced both mucosal and systemic antibody response in mice (61). The IN delivery of chitosan-delivered DNA vaccine against *Coxsackievirus* in mice enhanced the secretion of both serum IgG and mucosal IgA as well as CTLs activity in spleen (62). Consistent with the previous studies in mice (58–60, 63), the prime-boost vaccination of CNPs-KAg in pigs improved the IgA antibody secretion in the nasal passage and lungs. Importantly, robust secreted antibodies were cross-reactive against heterologous and heterosubtypic IAV and helped in significant reduction in nasal virus shedding and lung load of a heterologous challenge virus. In a previous experiment, PLGA-SwIAV KAg nanovaccine failed to reduce nasal virus shedding in spite of inducing a robust-specific cell-mediated immune response and reducing virus load in the lungs of most of the pigs. This anomaly was likely due to the inability of PLGA-encapsulated vaccine to induce mucosal IgA response (20). Similarly, polyanhydride-SwIAV KAg nanovaccine also enhanced specific cell-mediated immunity but did not enhance mucosal antibody responses and hence did not significantly reduce the nasal virus shedding (21). Like earlier murine studies (64, 65), IN vaccination with CNPs-KAg also induced influenza-specific systemic IgG antibody and HI titers.

Cell-mediated immunity is of prime importance for providing complete protection against intracellular pathogens. The Th1 cytokine IFN-γ is a critical cytokine involved in antiviral responses (66, 67). Chitosan is superior to alum adjuvant in enhancing the cell-mediated immune responses (68). It also induces type I IFN secretion from immature DCs which helps in DC maturation and generation of Th1-mediated cellular immune responses (69). In this study, enhanced IFNγ secretion by activated lymphocytes in a recall response with genetically variant IAVs was observed in both PBMCs and TBLN-MNCs of CNPs-KAg-vaccinated pigs. The observed spike in IFNγ recall response was associated with an enhanced virus-specific cellular response both at mucosal sites and systemically. Activated T cell subsets such as T helper and CTLs and innate NK cells are the sources of IFNγ (70). The prime-boost vaccination schedule employed in this study with CNPs-KAg increased the CTLs in PBMC cultures, the major source of IFNγ, the cytokine that helps in clearing virus from infected cells (67).

Another important T cell subset in pigs is T helper/memory cells (CD3^+^CD4^+^CD8α^+^) (71) which possesses cytolytic function and also secretes IFNγ. The protective response against pseudorabies virus infection has been attributed to the increased frequency of T helper/memory cells (71, 72). The frequency of T helper/memory cells in TBLN-MNCs was significantly enhanced in CNPs-KAg-vaccinated pigs. Thus, both T helper/memory and CTLs appear to contribute substantially in improving the cross-protective cellular immune response in pigs vaccinated with chitosan-based influenza nanovaccine.

In conclusion, the mucoadhesive chitosan-based IAV nanovaccine formulation delivered as IN mist augmented cross-reactive T and B lymphocytes response in pigs at both mucosal (upper and lower respiratory tracts and regional lymph nodes—TBLN) and systemic (blood) sites by augmenting secretary IgA, systemic IgG, and T cell responses against highly variant IAVs. This augmented virus-specific cross-reactive immune response resulted in a reduced nasal virus shedding, reduced viral titers in the pulmonary parenchyma, and relatively reduced inflammatory changes in the lungs. Thus, our study indicates that chitosan IAV nanovaccine might be an ideal vaccine candidate against constantly evolving influenza infections in swine herds. Future studies will focus on the optimization of CS:TPP:KAg combination to ensure a monodispersed nature, a higher positive charge, and better stability of CNPs-KAg vaccine. In our future vaccine challenge studies, the efficacy of IN CNPs-KAg vaccine will also be compared with commercial IM-killed and IN-modified live IAV vaccines, and in MDA positive piglets against variant field IAV isolates.

## Ethics Statement

This animal study was carried out in strict accordance with the recommendations by Public Health Service Policy, United States Department of Agriculture Regulations, the National Research Council’s Guide for the Care and Use of Laboratory Animals, and the Federation of Animal Science Societies’ Guide for the Care and Use of Agricultural Animals in Agricultural Research and Teaching. We followed all relevant institutional, state, and federal regulations and policies regarding animal care and use at The Ohio State University. All the pigs were maintained, samples collected, and euthanized, and all efforts were made to minimize the suffering of pigs. This study was carried out in accordance with the approved protocol of the Institutional Animal Care and Use Committee at The Ohio State University (Protocol number 2014A00000099).

## Author Contributions

SD and GR conceived and designed the project. SD, SR, CL, and GR performed the experiments, analyzed the data, and composed the manuscript. Vaccination trial in pigs, sample collection, and laboratory experiments were supported by SG, YL, BH, and NF-R. FL and HH worked on *in vitro* characterization of NPs. SK provided the pathological analysis of the pulmonary tissues of pigs. All the authors provided critical feedback on the manuscript prior to publication and have agreed to the final content.

## Conflict of Interest Statement

The authors declare that the research was conducted in the absence of any commercial or financial relationships that could be construed as a potential conflict of interest.
